# Exonuclease III (XthA) Enforces *In Vivo* DNA Cloning of Escherichia coli To Create Cohesive Ends

**DOI:** 10.1128/JB.00660-18

**Published:** 2019-02-11

**Authors:** Shingo Nozaki, Hironori Niki

**Affiliations:** a Microbial Genetics Laboratory, Genetic Strains Research Center, National Institute of Genetics, Mishima, Shizuoka, Japan; b Department of Genetics, The Graduate University for Advanced Studies (SOKENDAI), Mishima, Shizuoka, Japan; Indiana University Bloomington

**Keywords:** DNA cloning, DNA polymerase, *E. coli*, XthA, exonuclease, iVEC activity, linear DNA fragment, recombinant plasmid

## Abstract

Escherichia coli has an ability to assemble DNA fragments with homologous overlapping sequences of 15 to 40 bp at each end. Several modified protocols have already been reported to improve this simple and useful DNA cloning technology. However, the molecular mechanism by which E. coli accomplishes such cloning is still unknown. In this study, we provide evidence that the *in vivo* cloning of E. coli is independent of both RecA and RecET recombinases but is dependent on XthA, a 3′ to 5′ exonuclease. Here, *in vivo* cloning of E. coli by XthA is referred to as *in vivo* E. coli cloning (iVEC). We also show that iVEC activity is reduced by deletion of the C-terminal domain of DNA polymerase I (PolA). Collectively, these results suggest the following mechanism of iVEC. First, XthA resects the 3′ ends of linear DNA fragments that are introduced into E. coli cells, resulting in exposure of the single-stranded 5′ overhangs. Then, the complementary single-stranded DNA ends hybridize each other, and gaps are filled by DNA polymerase I. Elucidation of the iVEC mechanism at the molecular level would further advance the development of *in vivo* DNA cloning technology. Already we have successfully demonstrated multiple-fragment assembly of up to seven fragments in combination with an effortless transformation procedure using a modified host strain for iVEC.

**IMPORTANCE** Cloning of a DNA fragment into a vector is one of the fundamental techniques in recombinant DNA technology. Recently, an *in vitro* recombination system for DNA cloning was shown to enable the joining of multiple DNA fragments at once. Interestingly, E. coli potentially assembles multiple linear DNA fragments that are introduced into the cell. Improved protocols for this *in vivo* cloning have realized a high level of usability, comparable to that by *in vitro* recombination reactions. However, the mechanism of *in vivo* cloning is highly controversial. Here, we clarified the fundamental mechanism underlying *in vivo* cloning and also constructed a strain that was optimized for *in vivo* cloning. Additionally, we streamlined the procedure of *in vivo* cloning by using a single microcentrifuge tube.

## Abstract (precis)

Cloning of a DNA fragment into a vector is one of the fundamental techniques in recombinant DNA technology. Recently, an *in vitro* recombination system for DNA cloning was shown to enable the joining of multiple DNA fragments at once. Interestingly, E. coli potentially assembles multiple linear DNA fragments that are introduced into the cell. Improved protocols for this *in vivo* cloning have realized a high level of usability, comparable to that by *in vitro* recombination reactions. However, the mechanism of *in vivo* cloning is highly controversial. Here, we clarified the fundamental mechanism underlying *in vivo* cloning and also constructed a strain that was optimized for *in vivo* cloning. Additionally, we streamlined the procedure of *in vivo* cloning by using a single microcentrifuge tube.

## INTRODUCTION

Cloning of a DNA fragment into a vector is one of the fundamental techniques in recombinant DNA technology. A method using restriction enzymes and DNA ligases has long been used as the standard procedure for DNA cloning. Recently, modified methods of DNA cloning have been widely adopted in place of the canonical method. For example, for the joining of DNA fragments to vectors, an *in vitro* recombination reaction is used. In particular, enzymatic assembly of DNA fragments by using T5 exonuclease, DNA polymerase, and DNA ligase effectively joins multiple DNA fragments (1). T5 exonuclease resects the 5′ ends of the terminal overlapping sequences of the DNA fragments to create the 3′ ends of single-stranded DNA overhangs. The complementary single-stranded DNA overhangs are annealed, the gaps are filled, and the nicks are sealed enzymatically. A similar reaction also occurs with the crude cell extract of Escherichia coli (2, 3).

In contrast to DNA cloning utilizing *in vitro* recombination, some strains of E. coli can take up linear double-stranded vectors, insert DNA fragments, and assemble them *in vivo* (4, 5). The ends of these linear DNA fragments must contain 20 to 50 bp of overlapping homologous sequences. DNA amplification by PCR readily provides this type of linear DNA fragment of interest. Following its introduction in the early 1990s, this simpler cloning method was not widely used. Recently, however, it has been brought to scientific attention and has been improved with various strains of E. coli and several PCR-based protocols (6–12). These improved protocols for *in vivo* cloning have realized a high level of usability comparable to that by *in vitro* recombination reactions, since now it is only necessary to introduce PCR products into E. coli for the *in vivo* cloning.

The mechanism of *in vivo* cloning is highly controversial. Initially, the *sbcA23* mutant of the E. coli strain JC8679 was used for *in vivo* cloning, because the expression of RecE exonuclease and RecT recombinase, here referred to as RecET recombinase, of Rac prophage is activated in this mutant (5, 13). Then, it was thought that a recombination pathway of the prophage was involved in the *in vivo* cloning. However, E. coli strains without the *sbcA23* mutation, such as DH5α, also have sufficient ability for *in vivo* cloning (4, 8, 9). Recently, it was suggested that the ability for *in vivo* cloning is not limited to specific mutant strains (10, 11). If *in vivo* cloning is not dependent on host E. coli strains, then the DNA substrates may be responsible for the *in vivo* cloning. Klock et al. considered that the DNA fragments prepared by PCR have a single-stranded DNA region resulting from incomplete primer extension, and hybridization between complementary single-stranded ends promotes the pathway for *in vivo* cloning (6). On the other hand, Li et al. conjectured that 3′ to 5′ exonuclease activity of high-fidelity DNA polymerase creates a single-stranded region at the ends of the linear DNA fragments during PCR (7). Thus, the DNA fragments with single-stranded overhangs produced by PCR seem to be key for *in vivo* cloning. However, the linear DNA fragments prepared with a restriction enzyme that generates blunt ends are also capable of *in vivo* cloning, indicating that other mechanisms such as a gap repair reaction should be considered (8). In general, the mechanism of *in vivo* cloning remains unclear.

Here, we clarified the mechanism underlying the *in vivo* cloning of E. coli and also constructed an E. coli strain that was optimized for *in vivo* cloning. In addition, we streamlined the procedure of *in vivo* cloning by introducing a newly developed transformation procedure using a single microcentrifuge tube.

## RESULTS

### iVEC activity in various strains.

**FIG 1:**
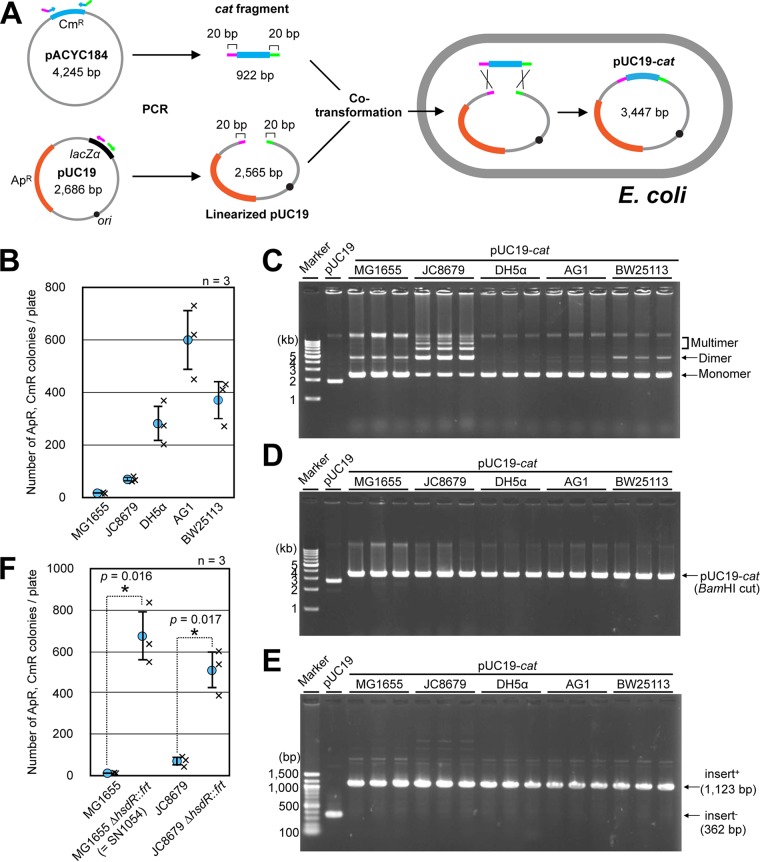
Assays of iVEC activities. (A) A scheme of *in vivo* cloning by assembly of two DNA fragments in a cell. DNA fragments containing the *cat* gene and linearized pUC19 DNA have 20-bp homologous overlapping ends (magenta and green). Ampicillin resistance (Ap^r^) and chloramphenicol resistance (Cm^r^) genes are shown in orange and light blue, respectively. (B) The iVEC activities of various strains are shown as the numbers of colonies resistant to both ampicillin and chloramphenicol. Averages from three independent experiments (×) are shown as circles with standard deviations. (C) Agarose gel electrophoresis of recombinant plasmids that were purified from the indicated strains. Plasmid DNA of pUC19 prepared from DH5α was used as a control. (D) Agarose gel electrophoresis of the plasmid DNA in panel C after digestion with BamHI. (E) Confirmation of insert DNA by PCR. The insert sequence was amplified by PCR, and the lengths of the PCR products were analyzed by agarose gel electrophoresis. pUC19 without an insert sequence was used as a negative control. (F) The iVEC activity of strains with Δ*hsdR* mutation. *, *P* values by Welch’s *t* test.

To identify the principle mechanism underlying the *in vivo* cloning in E. coli, here referred to as iVEC, we first confirmed the iVEC activity in various conventionally used strains of E. coli. We performed a simple assay of iVEC activity by transforming the strains with two DNA fragments that carry 20 bp of homologous overlaps at their ends: a *cat* gene encoding chloramphenicol acetyltransferase and the vector plasmid pUC19 (Fig. 1A). As a result, transformants resistant to both ampicillin and chloramphenicol appeared in all of the strains tested, although the efficiency of transformation varied depending on the host cells (Fig. 1B). Strains MG1655 and JC8679, in particular, had fewer transformants than the other strains. To confirm that the *cat* gene was cloned into pUC19, purified plasmids derived from the transformants were analyzed. All of the purified plasmids were larger than the empty vector, pUC19 (Fig. 1C). When the plasmids were digested with BamHI, a single band was detected in each lane, and the length of the band matched that of the cloned plasmid (Fig. 1D). The insertion of DNA into the vector was also confirmed by PCR (Fig. 1E).

Due to the smaller number of positive colonies in strains MG1655 and JC8679, we noticed that these strains have the wild-type *hsdR* gene. The three other strains, DH5α, AG1, and BW25113, have mutations in *hsdR*. HsdR is a host specificity restriction enzyme, which degrades DNA containing an unmethylated Hsd recognition sequence (14), and pUC19 DNA contains the recognition sequence. Therefore, we introduced a deletion mutation of the *hsdR* gene into MG1655 and JC8679, resulting in strains SN1054 and SN1071, respectively. As a result, the numbers of ampicillin- and chloramphenicol-resistant colonies after the introduction of both the *cat* fragment and linearized pUC19 were significantly increased by the deletion of *hsdR* (Fig. 1F). Thus, various E. coli strains essentially have the capacity to recombine short homologous sequences at the ends of linear DNAs, permitting the *in vivo* cloning of DNA fragments into linearized vectors.

### *recA* and *recET* are dispensable for iVEC activity.

**FIG 2:**
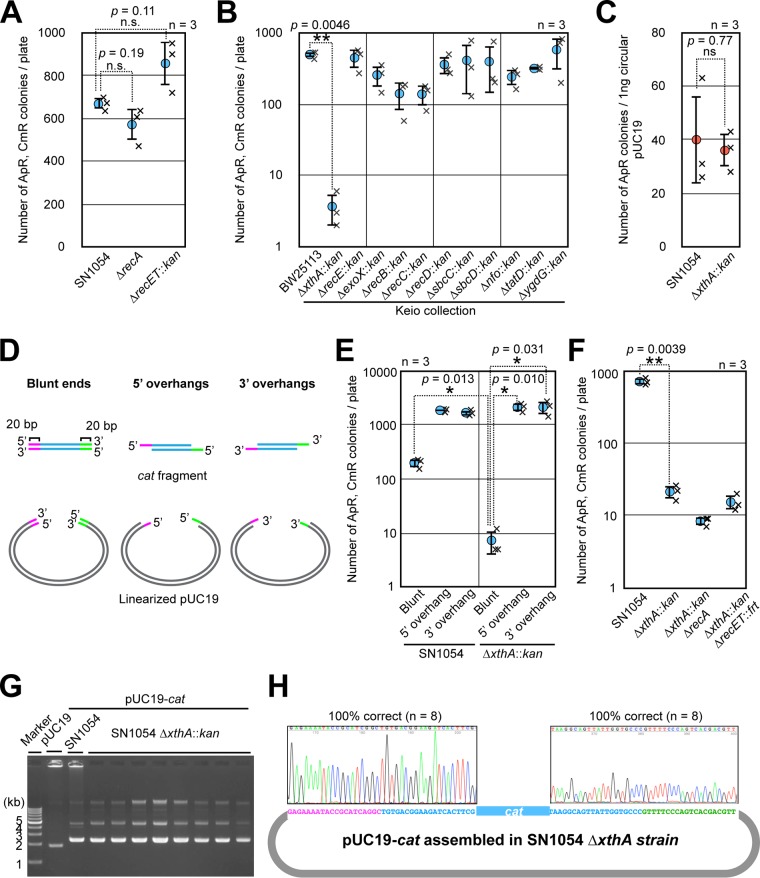
Effect of gene mutations on iVEC activities. (A) The iVEC activities of the Δ*recA* and Δ*recET* mutant strains are shown as the numbers of colonies resistant to both ampicillin and chloramphenicol. SN1054 was used as the wild-type strain. Averages from three independent experiments (×) are shown as circles with standard deviations. (B) The iVEC activities of single-gene deletion mutants for various exonucleases in the Keio collection. (C) Transformation efficiency of the Δ*xthA* strain. One nanogram of circular pUC19 DNA was used. Averages from three independent experiments (×) are shown as circles with standard deviations. (D) A diagram of DNA fragments with blunt ends, 5′ overhangs, and 3′ overhangs. *cat* fragments and linearized pUC19 have 20 bp of homologous sequences at ends (magenta and green). (E) iVEC activities by using DNA fragments with blunt ends, 5′ overhangs, and 3′ overhangs. These DNA fragments were introduced into SN1054 or the Δ*xthA* mutant. (F) The iVEC activities of double gene-deletion mutants: Δ*xthA* Δ*recA* and Δ*xthA* Δ*recET* mutants. (G) Plasmids assembled in the Δ*xthA* mutant strain were analyzed by agarose gel electrophoresis. pUC19 and pUC19-*cat* assembled in the *xthA*^+^ strain (SN1054) were used as a control. (H) Sequencing of the joint region of the plasmids assembled in the Δ*xthA* mutant strain. Eight plasmids of independent single colonies were analyzed. ns, not significant (*P* > 0.05); * or **, *P* values by Welch’s *t* test.

To elucidate the mechanism of iVEC activity in MG1655, we tested whether recombination proteins such as RecA or RecET were required for the *in vivo* cloning ability. For this purpose, we introduced deletion mutations of the *recA* or *recET* genes into strain SN1054. We then examined the iVEC activity by transforming these deletion mutants with the *cat* fragment and linearized pUC19. As a result, we found that deletion of *recA* or *recET* had little effect on iVEC activity (Fig. 2A), indicating that RecA and RecET are dispensable for *in vivo* cloning.

### *xthA* is required for iVEC activity.

In general, DNA recombination in E. coli accompanies the conversion of double-stranded DNA to single-stranded DNA by exonuclease. It is reported that E. coli has at least seven exonucleases that prefer double-stranded DNA for their substrates: XthA, RecE, ExoX, RecBCD, SbcCD, Nfo, and TatD (15). In addition, YgdG is an exonuclease whose preferential substrate is unknown. Therefore, we next examined the iVEC activity in exonuclease deletion mutants. We used the deletion mutants from the Keio collection, because BW25113, the parental strain of the Keio collection, has sufficient capacity for iVEC, as shown in Fig. 1B.

We tested each deletion mutant by introducing a DNA fragment containing the *cat* gene and linearized pUC19 vector. As a result, in the Δ*xthA* mutant, the iVEC activity was remarkably decreased to 0.7% of that in the wild-type strain (Fig. 2B). The iVEC activity was slightly decreased in the Δ*exoX*, Δ*recB*, Δ*recC*, Δ*nfo*, and Δ*tatD* mutants. However, because these defects were several orders of magnitude smaller than that observed in the Δ*xthA* mutant, we focused on XthA in the subsequent experiments.

There was a possibility that deficiency in plasmid maintenance or DNA uptake was the reason for the remarkable reduction of iVEC activity in the Δ*xthA* mutant. Therefore, we examined the level of transformation efficiency of the Δ*xthA* mutant by using circular DNA of the pUC19 plasmid and found that it was almost equivalent to the efficiency of the *xthA*^+^ strain (Fig. 2C). This indicates that plasmid maintenance and DNA uptake are normal in the Δ*xthA* strain. Since XthA (exonuclease III) has 3′ to 5′ exonuclease activity (16), we speculated that resection of the DNA ends by this enzyme to produce single-stranded overhangs is crucial for iVEC activity. To confirm this idea, we introduced DNA fragments, in which 20 bp of the single-stranded overhangs at the ends were generated in advance, into the Δ*xthA* mutant (Fig. 2D). As a result, in the Δ*xthA* mutant, a sufficient number of transformants comparable to the number in the *xthA*^+^ strain were obtained from the DNA fragments with overhangs, whereas DNA fragments with blunt ends yielded few recombinants (Fig. 2E). Hybridization between homologous single-stranded DNA regions of the introduced DNA fragments regardless of 5′ or 3′ overhangs would be essential for the recombination of the DNA fragments in the host cell. We concluded that the exonuclease activity of XthA to produce single-stranded overhangs plays a critical role in iVEC activity.

Although XthA was a major factor for iVEC activity, a small number of recombinant plasmids were still produced in the Δ*xthA* mutant (Fig. 2F). The transformants were obtained even when a mutation of Δ*recA* or Δ*recET* was added to the Δ*xthA* mutant. We confirmed that the recombinant plasmids were correctly assembled even in the Δ*xthA* mutant (Fig. 2G and H). Thus, faint iVEC activity still remained in the Δ*xthA* mutant. These results suggest that there are other minor pathways for iVEC activity, which are independent of XthA, RecA, and RecET.

### *polA* affects iVEC activity.

**FIG 3:**
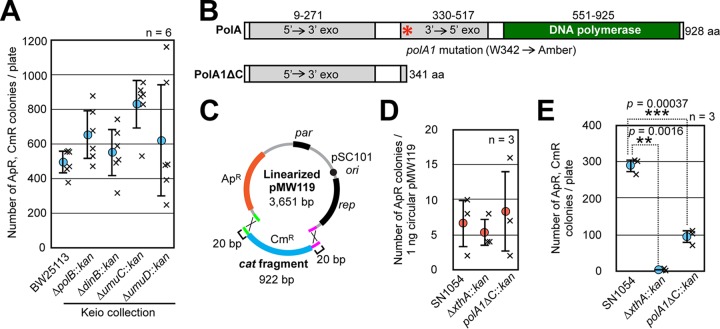
Involvement of DNA polymerases in iVEC activity. (A) The iVEC activities of various strains, which are deletion mutants of nonessential polymerases in the Keio collection, are shown as the numbers of colonies resistant to both ampicillin and chloramphenicol. Averages from six independent experiments (×) are shown as circles with standard deviations. (B) A diagram of functional domains in PolA and PolA1 polymerases. An asterisk indicates the point mutation site (W342 to amber) of *polA1* mutation. (C) Assembly of the *cat* fragment and linearized pMW119 is shown. Each fragment has 20 bp of homologous overlapping sequences shown in green and magenta. (D) Transformation efficiencies measured by using 1 ng of circular pMW119. Circles indicate averages with standard deviations from three independent experiments (×). (E) The iVEC activity of *polA1*ΔC is shown as the number of colonies resistant to both ampicillin and chloramphenicol after introduction of 0.15 pmol of the *cat* fragment and 0.05 pmol of linearized pMW119 into the indicated strains. Averages from three independent experiments (×) are shown as circles with standard deviations. ** or ***, *P* values versus the parent strain, SN1054, by Welch’s *t* test.

Our results suggested that, following the production of single-stranded DNA segments by XthA, homologous single-stranded DNA segments are hybridized and gaps are produced. We considered that specific DNA polymerases fill the gaps to ligate the hybridized DNA fragments. To address which DNA polymerase is involved in gap filling, we examined the effect of defects in DNA polymerases on iVEC activity. E. coli has five DNA polymerases (17). Among them, Pol II, Pol IV, and Pol V encoded by *polB*, *dinB*, and *umuCD*, respectively, are nonessential for cell growth. Therefore, we first tested the iVEC activities in the mutants with deletions of nonessential DNA polymerases. All of these deletions, i.e., Δ*polB*, Δ*dinB*, Δ*umuC*, and Δ*umuD*, had little effect on iVEC activity (Fig. 3A). Thus, these polymerases are not involved in iVEC activity.

Next, we examined the requirement of DNA polymerase I (Pol I) for iVEC activity. Pol I and Pol III are essential for cell growth. Pol III is a core enzyme of the DNA polymerase III holoenzyme, which is the primary enzyme complex involved in prokaryotic DNA replication. Hence, we considered that it would be difficult to analyze the iVEC activity by using a mutant of Pol III. On the other hand, although the *polA* gene encoding Pol I is required for cell growth on rich medium, the full length of this gene is not essential (18, 19). Only the N-terminal domain encoding 5′ to 3′ exonuclease is sufficient for cell growth (20). Indeed, a *polA1* mutant which expresses only 341 amino acid residues at the N terminus of PolA due to an amber mutation at amino acid residue 342 is viable (21) (Fig. 3B). Accordingly, we constructed a mutant strain harboring the *polA1* mutation along with a deletion of the part of the *polA* gene that encodes the C-terminal 587 amino acid residues, including the DNA polymerase domain. The resulting *polA1*ΔC mutant expresses the N-terminal 341 amino acid residues in the manner of the *polA1* mutant. Since the full-length PolA is required for the initiation step of pUC19 replication, we used pMW119 to assay iVEC activity (Fig. 3C). The replication origin of pMW119 is derived from pSC101, which does not require the *polA* product for the initiation of its replication (22). The transformation efficiencies of the *polA1*ΔC and Δ*xthA* mutants with pMW119 were similar to that of a wild-type strain, SN1054 (Fig. 3D). We measured the iVEC activity of SN1054 and the Δ*xthA* and *polA1*ΔC mutants by simultaneous introduction of linearized pMW119 and a DNA fragment containing the *cat* gene with 20-bp overlapping sequences at the ends. High iVEC activity was observed by using pMW119 in the wild-type strain but not in the Δ*xthA* mutant (Fig. 3E). Thus, *xthA* played a critical role in the iVEC activity when a pSC101-derivative plasmid vector was used. This result certainly suggests that application of iVEC is not limited to pUC derivative plasmids. The number of transformants of the *polA1*ΔC mutant decreased to about one-third of that of the wild-type strain, and this difference was statistically significant (*P = *0.00037 by Welch’s *t* test). In conclusion, the C-terminal domain of PolA was not fully responsible for, but did partly contribute to, the iVEC activity.

### Optimization of a host strain for iVEC.

**FIG 4:**
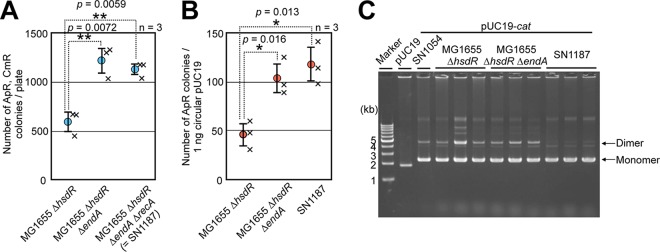
Construction of a strain optimized for iVEC. (A) Effects of Δ*hsdR* Δ*endA* and Δ*recA* on iVEC activities. The iVEC activities are shown as the numbers of colonies resistant to both ampicillin and chloramphenicol. Averages from three independent experiments (×) are shown as circles with standard deviations. (B) Transformation efficiencies measured by using 1 ng of circular pUC19 in each strain. Averages from three independent experiments (×) are shown as circles with standard deviations. (C) Agarose gel electrophoresis of recombinant plasmids (pUC19-*cat*). pUC19 was used as a control vector. The monomer and dimer of the plasmids are indicated by arrows. * or **, *P* values versus MG1655 Δ*hsdR* strain by Welch’s *t* test.

Since strains derived from MG1655 had the highest iVEC activity, we attempted to optimize the host strain based on MG1655. Many E. coli strains used for DNA manipulation, including DH5α, harbor a mutation in the *endA* gene, which encodes a DNA-specific endonuclease I (23) and improves the quantity of recovered plasmids. Therefore, we introduced a deletion mutation of the *endA* gene into E. coli strain MG1655 along with a deletion mutation of the *hsdR* gene. The number of positive colonies for iVEC increased by 2-fold in Δ*endA* cells compared to that of the *endA*^+^ strain (Fig. 4A). We examined the transformation efficiency of the Δ*endA* strain with pUC19 plasmid DNA and found that it was increased (Fig. 4B). This result indicates that the improvement in iVEC activity in the Δ*endA* strain was caused by increased transformation efficiency due to the DNA stability during the DNA uptake process.

In E. coli, dimer plasmid DNA accumulates due to homologous recombination (24). To prevent the dimerization of recombinant plasmids, we introduced a *recA* deletion mutation into a host strain carrying Δ*hsdR* Δ*endA* mutations, resulting in strain SN1187. Although the *recA* deletion mutation often causes lower transformation efficiency due to a reduction in cell viability, the iVEC activity and transformation efficiency of SN1187 were not deteriorated by the deletion mutation of *recA* (Fig. 4A and B). Moreover, the amount of dimer was drastically decreased when plasmid DNA was retrieved from SN1187 and analyzed by agarose gel electrophoresis (Fig. 4C).

### Multiple fragment cloning by the host strain SN1187.

**FIG 5:**
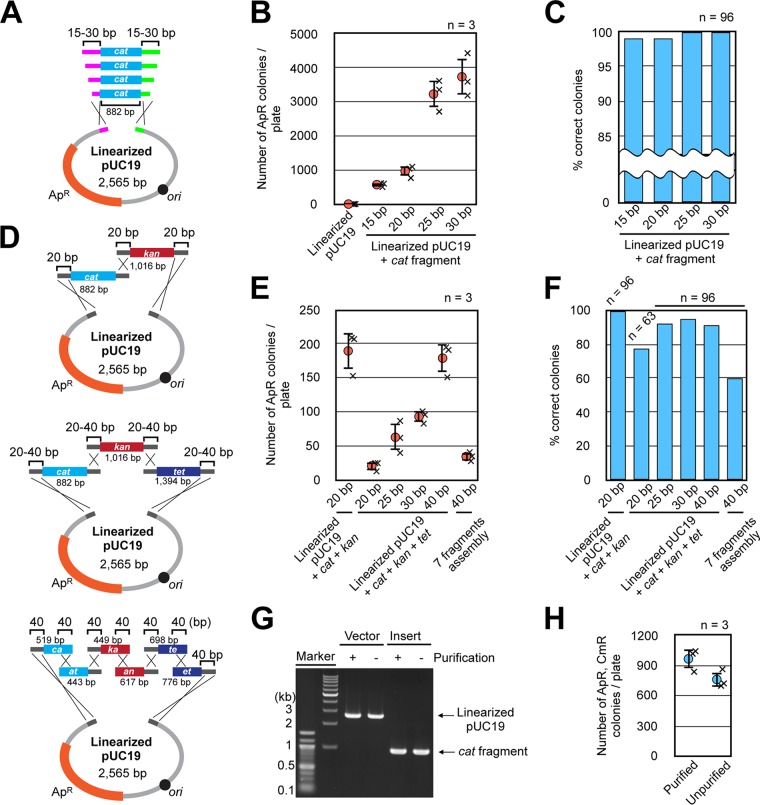
iVEC performance by the optimized strain. (A) A diagram of the assembly of two DNA fragments with various overlap lengths at the ends. (B) The iVEC activities by using two DNA fragments with various overlap lengths at the ends are shown as the numbers of colonies resistant to ampicillin. Averages from three independent experiments (×) are shown as circles with standard deviations. Introduction of only linearized pUC19 was also carried out as a negative control. (C) Proportions of colonies which were resistant to chloramphenicol among the 96 ampicillin-resistant colonies in panel B are shown as the percentages of correct colonies. (D) A diagram of the assembly of multiple DNA fragments with various overlap lengths at the ends. (E) The iVEC activities by using multiple DNA fragments with various overlap lengths at the ends are shown as the numbers of colonies resistant to ampicillin. Averages from three independent experiments (×) are shown as circles with standard deviations. (F) The proportions of colonies that were resistant to antibiotics among the 96 ampicillin-resistant colonies in panel E are shown as the percentages of correct colonies. Resistance to chloramphenicol and kanamycin was observed for the assembly of three fragments, and resistance to chloramphenicol, kanamycin, and tetracycline was observed for the assembly of four and seven fragments of ampicillin-resistant colonies (*n* = 96, except for assembly of the four DNA fragments with 20 bp overlaps [*n* = 63]). (G) Agarose gel electrophoresis of the PCR products with or without purification, which were used for the assembly of two fragments. (H) The iVEC activities by using the PCR products with or without purification are shown as the numbers of colonies resistant to ampicillin. The PCR products were DNA fragments with 20-bp overlaps at the ends. Averages from three independent experiments (×) are shown as circles with standard deviations.

We further evaluated the new host strain, SN1187, in terms of its capacity for iVEC. First, we examined whether certain lengths of homologous sequences at the ends of DNA fragments were required. We tested DNA fragments with overlapping sequences of 15 bp to 30 bp in length (Fig. 5A). In this experiment, the ampicillin-resistant colonies after introduction of both linearized pUC19 and the *cat* fragment were counted. Approximately 600, 1,000, 3,200, and 3,700 ampicillin-resistant colonies appeared when we used DNA fragments with overlapping sequences of 15 bp, 20 bp, 25 bp, and 30 bp at their ends, respectively (Fig. 5B). Most of the colonies (99% to 100%) were also resistant to chloramphenicol, indicating that the DNAs were correctly assembled in those colonies (Fig. 5C). On the other hand, when only linearized pUC19 was introduced, only 5 ampicillin-resistant transformants appeared (Fig. 5B). This result suggests that carryover of a small amount of template vector from PCR yielded few undesirable transformants, despite the fact that DpnI digestion of the template DNA from PCR was not carried out.

We also examined whether iVEC with SN1187 is available for multifragment assembly. First, we introduced three DNA fragments (linearized pUC19 and the DNA fragments including the *cat* or *kan* gene) with 20-bp overlapping sequences at their ends (Fig. 5D). Also in this experiment, we selected transformants with only ampicillin resistance, which is a marker of vector DNA, for practical purposes. As a result, approximately 200 ampicillin-resistant colonies were obtained (Fig. 5E). When we examined whether 96 randomly selected ampicillin-resistant colonies were also resistant to chloramphenicol and kanamycin, we found that all 96 colonies were resistant to chloramphenicol and kanamycin as well as ampicillin (Fig. 5F). Next, the assembly of four fragments (linearized pUC19 and the DNA fragments including the *cat*, *kan*, or *tet* gene) was carried out with 20 to 40 bp of homologous overlapping sequences (Fig. 5D). We obtained approximately 20, 60, 90, and 180 ampicillin-resistant colonies with homologous overlaps of 20, 25, 30, and 40 bp, respectively (Fig. 5E). The ratios of colonies resistant to ampicillin, chloramphenicol, kanamycin, and tetracycline against colonies resistant to ampicillin alone ranged from 80% to 95% (Fig. 5F). We also read joint sequences of assembled DNAs to confirm the accuracy of recombination. When 8 plasmids per construct of two-, three-, and four-fragment assemblies with 20-bp overlapping sequences were examined, no base change was found within overlapping sequences (see Fig. S1A to C in the supplemental material). Finally, we attempted a simultaneous gene assembly of seven fragments. Each of the DNA fragments used for the assembly of four fragments was split and assembled with 40-bp homologous overlaps at its ends (Fig. 5D). Approximately 40 colonies resistant to ampicillin were obtained (Fig. 5E). Among those ampicillin-resistant colonies, approximately 60% were also resistant to each of the antibiotics chloramphenicol, kanamycin, and tetracycline (Fig. 5F). This result indicated that the DNA fragments that included antibiotic resistance genes separated into 6 fragments were correctly assembled at the same time. We also examined joint sequences of this recombinant plasmid. For this purpose, plasmid DNA from 8 independent colonies was examined. While one plasmid had a 2-bp region of deletion within a joint segment, no base change was found in the other plasmids (Fig. S1D). Finally, we demonstrated that purification of the PCR products was not necessary for the iVEC activity. When unpurified PCR products were used directly for iVEC without PCR purification, the number of positive colonies was more than 500 (Fig. 5G and H). The PCR products can be used easily and relatively quickly without the requirement of any treatments such as column purification, ethanol precipitation, or DpnI digestion before transformation.

## DISCUSSION

**FIG 6:**
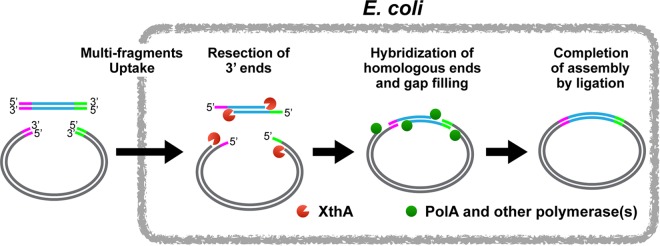
A model for the mechanism of iVEC.

XthA, also known as exodeoxyribonuclease III, exhibits 3′ to 5′ exonuclease activity. Introducing DNA fragments with cohesive ends into the E. coli cells effectively bypasses the requirement of XthA for the iVEC activity (Fig. 2E). On the other hand, the addition of cohesive ends to insert and vector DNA fragments also strengthens the iVEC activity in wild-type cells (Fig. 2E). This is consistent with previous reports that the generation of cohesive ends during PCR is effective for *in vivo* cloning (6, 7). Taken together, these facts indicate that the creation of cohesive ends from the blunt ends of DNA fragments is crucial for *in vivo* cloning. Therefore, we conclude that XthA exonuclease converts the blunt ends of double-stranded DNA to 5′ protruding ends in the process of *in vivo* cloning. In consideration of this activity, we propose the following as the most likely mechanism for iVEC as shown in Fig. 6. After the insert and the vector DNA fragments are introduced into the E. coli cell, XthA resects the ends of the DNA fragments from the 3′-to-5′ direction, producing 5′ overhanging ends. As the ends of insert and vector DNAs have mutually complementary sequences, the 5′ overhanging ends of the insert and the vector DNA fragments hybridize to each other as cohesive ends. In addition, the gaps are filled by DNA polymerases and the nicks are repaired by DNA ligases. Deletion of the DNA polymerase domain of PolA did not completely abrogate iVEC activity (Fig. 3E). There is a redundant polymerase(s) for the gap filling in iVEC. It is possible that Pol II, III, IV, or V is involved in the gap filling in the *polA1*ΔC background. Supposedly, Pol I is the major contributor, because DNA Pol I is the most abundant DNA polymerase in the E. coli cell.

Previously, a strain in which the expression of RecET recombinase was activated by a *sbcA23* mutation was used as a host strain for *in vivo* cloning (5). Therefore, it was thought that RecET was the recombinase essential for *in vivo* cloning. While strains without *sbcA23* mutations have been shown to possess iVEC activity (4, 8, 9), it was not clear whether even low-level expression of RecET was sufficient for iVEC. The present finding that the Δ*recET* mutant exhibited sufficient iVEC activity indicates that RecET is not required for iVEC (Fig. 2A). In addition, E. coli has other exonucleases in addition to XthA, but their contribution to the iVEC activity is relatively low (Fig. 2B). Interestingly, Δ*xthA* cells still maintained slight iVEC activity that was independent of *recA* or *recET* (Fig. 2F). This residual activity was not due to PCR-based production of single-stranded overhangs, since it was observed even in the assembly of DNA fragments with blunt ends (Fig. 2E). Thus, it seems likely that some other exonucleases are responsible for the residual iVEC activity in Δ*xthA* cells. XthA would be the dominant exonuclease that preferentially digests double-stranded DNA to produce single-stranded overhangs. Under most conditions, an E. coli strain having the exonuclease activity of XthA would be able to assemble DNA fragments with blunt ends that are generated by using conventional PCR.

Several derivatives of E. coli K-12 showed iVEC activity, suggesting that no specific mutations are required for iVEC activity. It seems likely that E. coli K-12 originally acquired iVEC activity, and the iVEC activity was involved in an unknown physiological function in E. coli. It is conceivable that XthA helps to repair minor DNA damage, instead of the RecBCD exonuclease. RecBCD produces a 3′ overhang and loads RecA onto the single-stranded DNA, causing an SOS response accompanied by cell division arrest (25). To help avoid such a serious outcome, it is conceivable that XthA functions in a repair pathway of DNA damage.

Linear DNA fragments introduced into the E. coli cell are usually degraded by the RecBCD exonuclease (26). However, multiple linear DNA fragments of iVEC escaped from the degradation of the RecBCD exonuclease. In this regard, the fact that linear DNA fragments are assembled by the action of XthA before RecBCD cleaves them may be due to a biased ratio between XthA and RecBCD. Liu et al. reported that the translation level of the *xthA* gene is approximately 50 times greater than that of *recBCD* (27).

In our present experiments, we found that the wild-type strain of E. coli exhibits iVEC activity (Fig. 4B). However, the level of this activity is unsatisfactory for DNA cloning. To improve the efficiency of iVEC, deletion mutations of *hsdR* and *endA* were introduced. The *hsdR* gene encodes a type I restriction enzyme, EcoKI (28), and EndA is a nonspecific DNA endonuclease (23). Both gene disruptions improved the transformation efficiency of the DNA fragments rather than the assembly process. It was expected that enhancement of the expression of *xthA* by using a T5/*lac* promoter would improve iVEC activity. However, we found that the enhanced expression did not increase iVEC activity.

We used a modified transformation and storage solution (TSS) method to measure iVEC activity. Cells in overnight culture were used to prepare competent cells for the measurement. Overnight-standing culture allows the entire process to be performed using only a single microcentrifuge tube, from the preparation of competent cells to transformation. A “one-tube” transformation protocol is sufficient for the TSS method but not for other methods of transformation. In the case of using exponentially growing cells, *in vivo* cloning can be performed by CaCl_2_ treatment or electroporation as well as the TSS method. However, the one-tube transformation protocol using overnight-standing culture worked only in the TSS method. The TSS method seems to be less affected by the growth phase. In this way, competent cells of many different strains can be easily prepared. However, the transformation of plasmid DNA is not very high: approximately 10^4^ to 10^5^ CFU/µg pUC19 (Fig. 4B). Therefore, by using less than 10 to 100 pg of template plasmid in PCR products, the background of unwanted vector-only colonies can be significantly reduced. This also means that DpnI treatment after PCR with vector DNA is dispensable for reducing transformants by the template plasmid DNA. In fact, we were able to obtain the desired colonies despite a lower number of transformants. The number of positive transformants obtained with iVEC using our method and the host strain, SN1187, is comparable or greater than that in previous reports using other methods, such as the rubidium chloride method or commercially available competent cells.

E. coli cells can simultaneously uptake multiple DNA fragments via an unknown mechanism. As a result, the assembly of up to seven fragments was possible via iVEC (Fig. 5D to F). In addition, this approach was effective for obtaining recombinant products of less than 10 kbp in total. To hybridize the cohesive ends of DNA fragments, shorter DNA fragments would be suitable because the opportunity for initial contact between the ends of the DNA fragments increases. Our procedure could be utilized for multisite-directed mutagenesis instead of primer extension mutagenesis. Furthermore, an improved understanding of iVEC activity may contribute to the development of iVEC methods in the future.

## MATERIALS AND METHODS

### Medium.

L broth (1% Difco tryptone, 0.5% Difco yeast extract, 0.5% NaCl, pH adjusted to 7.0 with 5 N NaOH) was used for liquid culture. The agar plate was made of L broth and 1.5% agar. The following antibiotics were used as needed: 50 µg/ml of ampicillin, 10 µg/ml of chloramphenicol, 15 µg/ml of kanamycin, and 10 µg/ml of tetracycline.

### Bacterial strains and plasmids.

The E. coli strains and plasmids used in this work are listed in Tables S1 and S2 in the supplemental material, respectively. To construct a Δ*hsdR*::*frt* mutant, a chromosomal DNA segment containing Δ*hsdR*::*kan* was amplified from genomic DNA of the Δ*hsdR*::*kan* strain in the Keio collection by PCR using the primer set hsdR_F/hsdR_R (29). The amplified DNA fragments were introduced into the parent strains with pKD46 as described by Datsenko and Wanner (30). The Δ*xthA*::*kan*, Δ*recET*::*kan*, and *polA1*ΔC::*kan* strains were constructed in a similar manner using the primer sets and templates xthA_F/xthA_R and chromosome of Keio Δ*xthA*::*kan*, recET_F/recET_R and pKD4, and polAdelC_F/polAdelC_R and pKD4, respectively. The *kan* cassette was removed by pCP20, if needed (30). To construct a Δ*recA* strain, plasmid DNA of pKH5002SB was amplified by using the primer set pKH_F/pKH_R. Upstream and downstream chromosomal segments of the *recA* gene were amplified from MG1655 genomic DNA by using the primer sets recAup_F/recAup_R and recAdown_F/recAdown_R. We obtained a 1.8-kb upstream chromosomal segment and a 2-kb downstream chromosomal segment of *recA*, respectively. Both the recAup_F primer and the recAdown_R primer have an additional 20-bp sequence complementary to primers pKH_R and pKH_F, respectively. In addition, 40 bp of the sequences within the primers recAup_R and recAdown_F are complementary to each other. Amplified DNA fragments of pKH5002SB and the upstream and downstream regions of a chromosomal segment of *recA* were introduced into a Δ*rnhA*::*kan* strain to generate pKH5002SBΔ*recA* (Fig. S2A). Using this plasmid, the *recA* gene was deleted with two successive homologous recombinations as described previously (31) (Fig. S2B). The Δ*hsdR* and Δ*endA* strains were constructed by using the same method with the primer sets hsdRup_F/hsdRup_R and hsdRdown_F/hsdRdown_R and endAup_F/endAup_R/endAdown_F/endAdown_R, respectively.

### Preparation of PCR products for transformation.

We used KOD plus Neo (Toyobo) for PCR. The thermal cycler program was as follows: 94°C for 2 min, followed by 30 cycles of 98°C for 10 s, 58°C for 10 s, and 68°C for 30 s/kb, and a final extension of 68°C for 5 min. The oligonucleotide primers used for PCR are listed in Tables S3 and S4. The final concentration of the template DNA in each reaction mixture was adjusted to 1 pg/µl, e.g., 50 pg in a 50-µl reaction mixture. The *cat* (chloramphenicol resistance) and *tet* (tetracycline resistance) genes were amplified from pACYC184 DNA, and the *kan* (kanamycin resistance) gene was amplified from pACYC177 DNA. All PCR products were purified using a Wizard SV PCR cleanup system (Promega). Digestion of template DNA by DpnI was not necessary after PCR.

### Preparation of DNA fragments with blunt ends, 5′ overhangs, or 3′ overhangs.

DNA fragments with blunt ends, 5′ overhangs, or 3′ overhangs were prepared as follows. To isolate single-stranded strands, we used a Long ssDNA Preparation kit (BioDynamics Laboratory, Tokyo). Plasmids used for the isolation of ssDNAs are listed in Table S2 . Each pair of the top and the bottom single-stranded DNA fragments for blunt ends, 5′ overhangs, or 3′ overhangs was mixed and incubated at 99°C for 5 min and annealed at 65°C for 30 min to generate double-stranded DNA.

### Transformation.

To introduce DNA fragments into E. coli cells, we used the TSS method with modifications (32). A small number of cells in a colony on an agar plate were picked up using a sterilized toothpick and suspended in a 1.5-ml microcentrifuge tube containing 1 ml of L broth. The tube lid was closed. The tube was standing in an incubator at 37°C for 20 h without shaking. After standing incubation for 20 h, the optical density at 600 nm (OD_600_) of the culture reached approximately 1.4, and the number of cells in the tube was approximately 4 × 10^8^ CFU/ml. The tube was chilled on ice for 10 min and centrifuged at 5,000 × *g* for 1 min at 4°C to spin down the cells. The supernatant was removed, and the cell pellet was dissolved in 100 µl of ice-cold TSS (50% L broth, 40% 2× TSS, and 10% dimethyl sulfoxide [DMSO]) mixed with DNA. The composition of the 2× TSS was 20% (wt/vol) polyethylene glycol 8000 (PEG 8000), 100 mM MgSO_4_, and 20% (vol/vol) glycerol in L broth. For DNA cloning, 0.05 pmol of linearized vector and 0.15 pmol of each insert DNA fragment were used. After gentle mixing, the solution was immediately frozen in liquid nitrogen for 1 min. Frozen tubes were transferred to an ice bath. After a 10-min incubation on ice, the tubes were briefly vortexed to mix their contents and incubated on ice for an additional 10 min. Then, 1 ml of L broth was added, and the contents of the tube were mixed by inversion and incubated at 37°C for 45 min. After incubation, the cells were centrifuged, and the supernatant was roughly discarded. The cell pellet was dissolved in the remaining supernatant, and the cell suspension was spread on an L agar plate containing appropriate antibiotics. Finally, the plates were incubated at 37°C for 16 h, and the colonies were counted. To examine transformation efficiency, 1 ng of the indicated circular plasmids was used.

### Assay of iVEC activity.

A DNA fragment containing an antibiotic resistance gene and linearized pUC19 with 20-bp homologous overlapping ends were amplified by PCR and introduced into E. coli cells according to the modified TSS method described above (Fig. 1A). In a standard assay of the iVEC activity, 0.15 pmol of the *cat* fragment and 0.05 pmol of linearized pUC19 were used for the transformation of indicated strains. We counted the colonies resistant to both ampicillin and chloramphenicol after simultaneous introduction of the *cat* fragment and linearized pUC19 into indicated strains.

## Supplementary Material

Supplemental file 1
