# Supplementary material for: Exonuclease III (XthA) Enforces In Vivo DNA Cloning of Escherichia coli To Create Cohesive Ends
Source: J Bacteriol. 2019 Feb 11;201(5):e00660-18. doi: 10.1128/JB.00660-18 (PMC6379578; doi:10.1128/JB.00660-18)
Supplement: Supplemental file 1 [file 844b34b269503ec7f906b6c17357aee3_JB.00660-18-s0001.pdf]

Table S1: Strains used in this work

Strain	Parent strain and Genotype	Source or Reference
MG1655	F-, *rph-1*	NBRP *E. coli*, Japan
JC8679	F-, *thr-1*, *leuB6*, *thi-1*, *lacY1*, *galK2*, *ara-4*, *xyl-5*, *mtl-1*, *proA2*, *his-60*, *argE3*, *rpsL31*, *tsx-33*, *supE44*, *recB21*, *recC22*, *sbcA23*	Gillen *et al*., 1981
DH5α	F-, *deoR*, *endA1*, *gyrA96*, *hsdR17*(rK-, mK+), *recA1*, *relA1*, *supE44*, *thi-1*, Δ(*lacZYA- argF*)U169, (Phi80*lacZ*ΔM15)	NBRP *E. coli*, Japan
AG1	F-, *recA1*, *endA1*, *gyrA96*, *thi-1*, *hsdR17*(rK-, mK+), *supE44*, *relA1*	Kitagawa *et al*., 2005
BW25113	F-, *rrnB*, Δ*lacZ*4787, *hsdR514*, Δ*araBAD*567, Δ*rhaBAD*568, *rph-1*	Datsenko and Wanner, 2000
SN1054	MG1655 Δ*hsdR*::*frt*	This work
SN1071	JC8679 Δ*hsdR*::*frt*	This work
SN1194	SN1054 Δ*recA*(Δ2,820,759-2,821,785)	This work
SN1097	SN1054 Δ*recET*::*kan*	This work
SN1077	SN1054 Δ*xthA*::*kan*	This work
SN1203	SN1054 Δ*xthA*::*kan* Δ*recA*(Δ2,820,759-2,821,785)	This work
SN1201	SN1054 Δ*recET*::*frt* Δ*xthA*::*kan*	This work
SN1085	SN1054 *polA*ΔC::*kan*	This work
SN1146	MG1655 Δ*hsdR*(Δ4,581,454-4,584,720)	This work
SN1171	MG1655 Δ*hsdR* Δ*endA*(Δ3,088,303-3,089,037)	This work
SN1187	MG1655 Δ*hsdR* Δ*endA* Δ*recA*(Δ2,820,759-2,821,785)	This work
JW1738-KC	BW25113 Δ*xthA*::*kan*	Baba *et al*., 2006
JW1344-KC	BW25113 Δ*recE*::*kan*	Baba *et al*., 2006
JW1833-KC	BW25113 Δ*exoX*::*kan*	Baba *et al*., 2006
JW2788-KC	BW25113 Δ*recB*::*kan*	Baba *et al*., 2006
JW2790-KC	BW25113 Δ*recC*::*kan*	Baba *et al*., 2006
JW2787-KC	BW25113 Δ*recD*::*kan*	Baba *et al*., 2006
JW0387-KC	BW25113 Δ*sbcC*::*kan*	Baba *et al*., 2006
JW0388-KC	BW25113 Δ*sbcD*::*kan*	Baba *et al*., 2006
JW2146-KC	BW25113 Δ*nfo*::*kan*	Baba *et al*., 2006
JW5931-KC	BW25113 Δ*tatD*::*kan*	Baba *et al*., 2006

JW5446-KC	BW25113 Δ*ygdG*::*kan*	Baba *et al*., 2006
JW0059-KC	BW25113 Δ*polB*::*kan*	Baba *et al*., 2006
JW0221-KC	BW25113 Δ*dinB*::*kan*	Baba *et al*., 2006
JW1173-KC	BW25113 Δ*umuC*::*kan*	Baba *et al*., 2006
JW1172-KC	BW25113 Δ*umuD*::*kan*	Baba *et al*., 2006
JW4313-KC	BW25113 Δ*hsdR*::*kan*	Baba *et al*., 2006
JW0204-KC	BW25113 Δ*rnhA*::*kan*	Baba *et al*., 2006

Deletion positions of Δ*recA*, Δ*hsdR* and Δ*endA* are described according to the genome sequence of MG1655 (U00096.3).

The names of strains in the Keio collection are described as JWxxxx-KC according to the designation in NBRP *E. coli*, Japan.

Table S2: Plasmids

Name	Reference or source	Comments
pUC19	NBRP *E. coli*, Japan	
pACYC184	NBRP *E. coli*, Japan	
pACYC177	NBRP *E. coli*, Japan	
pMW119	Nippon Gene Co., Ltd.	
pKH5002SB	Kitagawa *et al*., 1998	
pKH5002SB-Δ*hsdR*	This work	
pKH5002SB-Δ*endA*	This work	
pKH5002SB-Δ*recA*	This work	
pKD4	Datsenko and Wanner, 2000	For amplification of the *kan* cassette.
pKD46	Datsenko and Wanner, 2000	For expression of lambda Red recombinase.
pCP20	Datsenko and Wanner, 2000	For expression of flippase (FLP) recombinase.
pLSODN-4D	BioDynamics Laboratory Inc.	Long the ssDNA Preparation Kit.
pLSODN4D-*cat*T	This work	For preparation of the top strand ssDNA of the *cat* fragment with blunt ends, a *cat* fragment in which the BsrDI site [GCAATG] was mutated to [GCgATG] was cloned into the site between Nt.BspQI and Nb.BsrDI of pLSODN-4D.
pLSODN4D-*cat*B	This work	For preparation of the bottom strand ssDNA of the *cat* fragment with blunt ends, a *cat* fragment with the mutated BsrDI site was cloned into the site between Nt.BspQI and Nb.BsrDI of pLSODN-4D in the direction opposite that used for pLSODN4D-catT.
pLSODN4D-pUCT	This work	For preparation of the top strand ssDNA of the linearized pUC19 fragment with blunt ends, a linearized pUC19 fragment in which the BspQI site [GCTCTTC] and BsrDI sites [GCAATG] were mutated to [GCTgTTC] and [GCgATG], respectively, was cloned into the site between Nt.BspQI and Nb.BsrDI of pLSODN-4D.

pLSODN4D-pUCB	This work	For preparation of the bottom strand ssDNA of the linearized pUC19 fragment with blunt ends, a linearized pUC19 fragment with the mutated BspQI and BsrDI sites was cloned into the site between Nt.BspQI and Nb.BsrDI of pLSODN-4D in the direction opposite that used for pLSODN4D-pUCT.
pLSODN4D-*cat*5'T	This work	For preparation of the top strand ssDNA of the *cat* fragment with 5' overhangs, a *cat* fragment with a mutated BsrDI site and 20 bp of deletion at the 3' end was cloned into the site between Nt.BspQI and Nb.BsrDI of pLSODN-4D.
pLSODN4D-*cat*5'B	This work	For preparation of the bottom strand ssDNA of the *cat* fragment with 5' overhangs, a *cat* fragment with a mutated BsrDI site and 20 bp of deletion at the 5' end was cloned into the site between Nt.BspQI and Nb.BsrDI of pLSODN-4D in the direction opposite that used for pLSODN4D-cat5'T.
pLSODN4D-pUC5'T	This work	For preparation of the top strand ssDNA of the linearized pUC19 fragment with 5' overhangs, a linearized pUC19 fragment with mutated BspQI and BsrDI sites and 20 bp of deletion at the 3' end was cloned into the site between Nt.BspQI and Nb.BsrDI of pLSODN-4D.
pLSODN4D-pUC5'B	This work	For preparation of the bottom strand ssDNA of the linearized pUC19 fragment with 5' overhangs, a linearized pUC19 fragment with mutated BspQI and BsrDI sites and 20 bp of deletion at the 5' end was cloned into the site between Nt.BspQI and Nb.BsrDI of pLSODN-4D in the direction opposite that used

		for pLSODN4D-pUC5'T.
pLSODN4D- *cat*3'T	This work	For preparation of the top strand ssDNA of the *cat* fragment with 3' overhangs, a *cat* fragment with a mutated BsrDI site and 20 bp of deletion at the 5' end was cloned into the site between Nt.BspQI and Nb.BsrDI of pLSODN-4D.
pLSODN4D-*cat*3'B	This work	For preparation of the bottom strand ssDNA of the *cat* fragment with 3' overhangs, a *cat* fragment with a mutated BsrDI site and 20 bp of deletion at the 3' end was cloned into the site between Nt.BspQI and Nb.BsrDI of pLSODN-4D in the direction opposite that used for pLSODN4D-cat3'T.
pLSODN4D-pUC3'T	This work	For preparation of the top strand ssDNA of the linearized pUC19 fragment with 3' overhangs, a linearized pUC19 fragment with mutated BspQI and BsrDI sites and 20 bp of deletion at the 5' end was cloned into the site between Nt.BspQI and Nb.BsrDI of pLSODN-4D.
pLSODN4D-pUC3'B	This work	For preparation of the bottom strand ssDNA of the linearized pUC19 fragment with 3' overhangs, a linearized pUC19 fragment with mutated BspQI and BsrDI sites and 20 bp of deletion at the 3' end was cloned into the site between Nt.BspQI and Nb.BsrDI of pLSODN-4D in the direction opposite that used for pLSODN4D-pUC3'T.

Table S3: Oligonucleotide Primers used for PCR

Name	Oligonucleotide sequence (5'-3')
pUC_F	GTTTTCCCAGTCACGACGTT
pUC_R	GCCTGATGCGGTATTTTCTC
pUC(tet20)_F	GAGAACTGTGAATGCGCAAA GTTTTCCCAGTCACGACGTT
pUC(cat20)_R	CGAAGTGATCTTCCGTCACA GCCTGATGCGGTATTTTCTC
cat(pUC15)_F	AATACCGCATCAGGC TGTGACGGAAGATCACTTCG
cat(pUC20)_F	GAGAAAATACCGCATCAGGC TGTGACGGAAGATCACTTCG
cat(pUC25)_F	GTAAGGAGAAAATACCGCATCAGGC TGTGACGGAAGATCACTTCG
cat(pUC30)_F	GATGCGTAAGGAGAAAATACCGCATCAGGC TGTGACGGAAGATCACTTCG
cat(pUC15)_R	CGTGACTGGGAAAAC GGGCACCAATAACTGCCTTA
cat(pUC20)_R	AACGTCGTGACTGGGAAAAC GGGCACCAATAACTGCCTTA
cat(pUC25)_R	TTTACAACGTCGTGACTGGGAAAAC GGGCACCAATAACTGCCTTA
cat(pUC30)_R	GTCGTTTTACAACGTCGTGACTGGGAAAAC GGGCACCAATAACTGCCTTA
cat(kan10)_R	GGCTTTGTTG GGGCACCAATAACTGCCTTA
cat(kan15)_R	AACGTGGCTTTGTTG GGGCACCAATAACTGCCTTA
cat(kan20)_R	GACACAACGTGGCTTTGTTG GGGCACCAATAACTGCCTTA
kan(cat10)_F	ATTGGTGCCC CAACAAAGCCACGTTGTGTC
kan(cat15)_F	CAGTTATTGGTGCCC CAACAAAGCCACGTTGTGTC
kan(cat20)_F	TAAGGCAGTTATTGGTGCCC CAACAAAGCCACGTTGTGTC
kan(pUC20)_R	AACGTCGTGACTGGGAAAAC TCCCGTCAAGTCAGCGTAAT
kan(tet10)_R	GCTGACTTCA TCCCGTCAAGTCAGCGTAAT
kan(tet15)_R	ATGGGGCTGACTTCA TCCCGTCAAGTCAGCGTAAT
kan(tet20)_R	ATCGTATGGGGCTGACTTCA TCCCGTCAAGTCAGCGTAAT
tet(kan10)_F	CTTGACGGGA TGAAGTCAGCCCCATACGAT
tet(kan15)_F	GCTGACTTGACGGGA TGAAGTCAGCCCCATACGAT
tet(kan20)_F	ATTACGCTGACTTGACGGGA TGAAGTCAGCCCCATACGAT
tet(pUC20)_R	AACGTCGTGACTGGGAAAAC TTTGCGCATTCACAGTTCTC
tet(pUC25)_R	TTTACAACGTCGTGACTGGGAAAAC TTTGCGCATTCACAGTTCTC
tet(pUC30)_R	GTCGTTTTACAACGTCGTGACTGGGAAAAC TTTGCGCATTCACAGTTCTC
pUC_check_F	CGGCATCAGAGCAGATTGTA
pUC_check_R	TGTGGAATTGTGAGCGGATA

pMW_F	GCAGAGCGAGGTATGTAGGC
pMW_R	CTGGCGTAATAGCGAAGAGG
cat(pMW)_F	CCTCTTCGCTATTACGCCAG TGTGACGGAAGATCACTTCG
cat(pMW)_R	GCCTACATACCTCGCTCTGC GGGCACCAATAACTGCCTTA
catN_R	CGTTTCAGTTTGCTCATGGA
catC_F	TCACCCTTGTTACACCGTTT
kanN_R	TGTTTTCCCGGGGATCGCAG
kanC_F	TGATGCATGGTTACTCACCA
tetN_R	CTCCCTTATGCGACTCCTGC
tetC_F	CTACTGGGCTGCTTCCTAAT
pUC_seq_F	CGGTGAAAACCTCTGACACA
cat_seq_F	TACACCGTTTTCCATGAGCA
kan_seq_F	GGTTGCATTCGATTCCTGTT
tet_seq_F	GGCAGGTAGATGACGACCAT
hsdR_F	CCAGCCTGCACGATTTTTAT
hsdR_R	GCTGACCAGTGCGGTTATTT
recET_F	ATCATTCACTGAACAAAACGAATTTTAATCTGAGTTGAGGTTAAAAAACA GTGTAGGCTGGAGCTGCTTC
recET_R	TCTCATAAAAAATATTTCAAGTTGGCGGTGCATTACACCGCCAGGCTGAA CATATGAATATCCTCCTTAG
xthA_F	AACAACAGGCGGTAAGCAAC
xthA_R	AGTTTGAGCCAGGAGAGCTG
polAdelC_F	CTTATGACAACTACGTCACCATCCTTGATGAAGAAACACTGAAAGCGTAG GTGTAGGCTGGAGCTGCTTC
polAdelC_R	GTGACAGCTTATGTTGCTTACTTACGAAAAAAGGCATGTTCAGGCGAATC CATATGAATATCCTCCTTAG
pKH_F	AGCTCACTCAAAGGCGGTAA
pKH_R	TTATGCCCATGCAACAGAAA
hsdRup_F	TTACCGCCTTTGAGTGAGCT TGCTGGACAATCTCGACAAG
hsdRup_R	AAACGATCGAGCCAGCTTAA GGCGATGGCATAAGTGAAGT
hsdRdown_F	ACTTCACTTATGCCATCGCC TTAAGCTGGCTCGATCGTTT
hsdRdown_R	TTTCTGTTGCATGGGCATAA AACTCGGCATATTGGTACGC

endAup_F	TTTCTGTTGCATGGGCATAA ACGCAAGAATGGGTCGTAAG
endAup_R	GTTATGATTGCCCTGCACCT TTGGTATGCAATCAGCCAGA
endAdown_F	TCTGGCTGATTGCATACCAA AGGTGCAGGGCAATCATAAC
endAdown_R	TTACCGCCTTTGAGTGAGCT TTCGATGGCATCCATTAACA
recAup_F	TTTCTGTTGCATGGGCATAA TACTTGCCCCTGGTTGAATC
recAup_R	GCTACGCCTTCGCTATCATC TCCTGTCATGCCGGGTAATA
recAdown_F	TATTACCCGGCATGACAGGA GATGATAGCGAAGGCGTAGC
recAdown_R	TTACCGCCTTTGAGTGAGCT CCAGAAAGTTTTGCCAGCTC

Underlining indicates the added overlapping sequences.

Table S4: Primer sets used for PCR

PCR product	Primer set
Linearized pUC19	pUC_F and pUC_R
cat fragment with 15 bp of overlaps with linearized pUC19	cat(pUC15)_F and cat(pUC15)_R
cat fragment with 20 bp of overlaps with linearized pUC19	cat(pUC20)_F and cat(pUC20)_R
cat fragment with 25 bp of overlaps with linearized pUC19	cat(pUC25)_F and cat(pUC25)_R
cat fragment with 30 bp of overlaps with linearized pUC19	cat(pUC30)_F and cat(pUC30)_R
cat fragment with 20 bp of overlaps for assembly of 3 fragments	cat(pUC20)_F and cat(kan10)_R
kan fragment with 20 bp of overlaps for assembly of 3 fragments	kan(cat10)_F and kan(pUC20)_R
cat fragment with 20 bp of overlaps for assembly of 4 fragments	cat(pUC20)_F and cat(kan10)_R
kan fragment with 20 bp of overlaps for assembly of 4 fragments	kan(cat10)_F and kan(tet10)_R
tet fragment with 20 bp of overlaps for assembly of 4 fragments	tet(kan10)_F and tet(pUC20)_R
cat fragment with 25 bp of overlaps for assembly of 4 fragments	cat(pUC25)_F and cat(kan15)_R
kan fragment with 25 bp of overlaps for assembly of 4 fragments	kan(cat10)_F and kan(tet15)_R
tet fragment with 25 bp of overlaps for assembly of 4 fragments	tet(kan10)_F and tet(pUC25)_R
cat fragment with 30 bp of overlaps for assembly of 4 fragments	cat(pUC30)_F and cat(kan15)_R
kan fragment with 30 bp of overlaps for assembly of 4 fragments	kan(cat15)_F and kan(tet15)_R
tet fragment with 30 bp of overlaps for assembly of 4 fragments	tet(kan15)_F and tet(pUC30)_R
Linearized pUC19 for assembly of 4 or 7 fragments	pUC(tet20)_F and pUC(cat20)_R
cat fragment with 40 bp of overlaps for assembly of 4 fragments	cat(pUC20)_F and cat(kan20)_R
kan fragment with 40 bp of overlaps for assembly of 4 fragments	kan(cat20)_F and kan(tet20)_R
tet fragment with 40 bp of overlaps for assembly of 4 fragments	tet(kan20)_F and tet(pUC20)_R
The first half of split *cat* fragment with 40 bp of overlaps	cat(pUC20)_F and catN_R
The latter half of split *cat* fragment with 40 bp of overlaps	catC_F and cat(kan20)_R
The first half of split *kan* fragment with 40 bp of overlaps	kan(cat20)_F and kanN_R
The latter half of split *kan* fragment with 40 bp of overlaps	kanC_F and kan(tet20)_R
The first half of split *tet* fragment with 40 bp of overlaps	tet(kan20)_F and tetN_R
The latter half of split *tet* fragment with 40 bp of overlaps	tetC_F and tet(pUC20)_R
Linearized pMW119	pMW_F and pMW_R
cat fragment with 20 bp of overlaps for assembly with pMW119	cat(pMW)_F and cat(pMW)_R
Confirmation of the insert sequence in pUC19	pUC_check_F and pUC_check_R

Figure S1

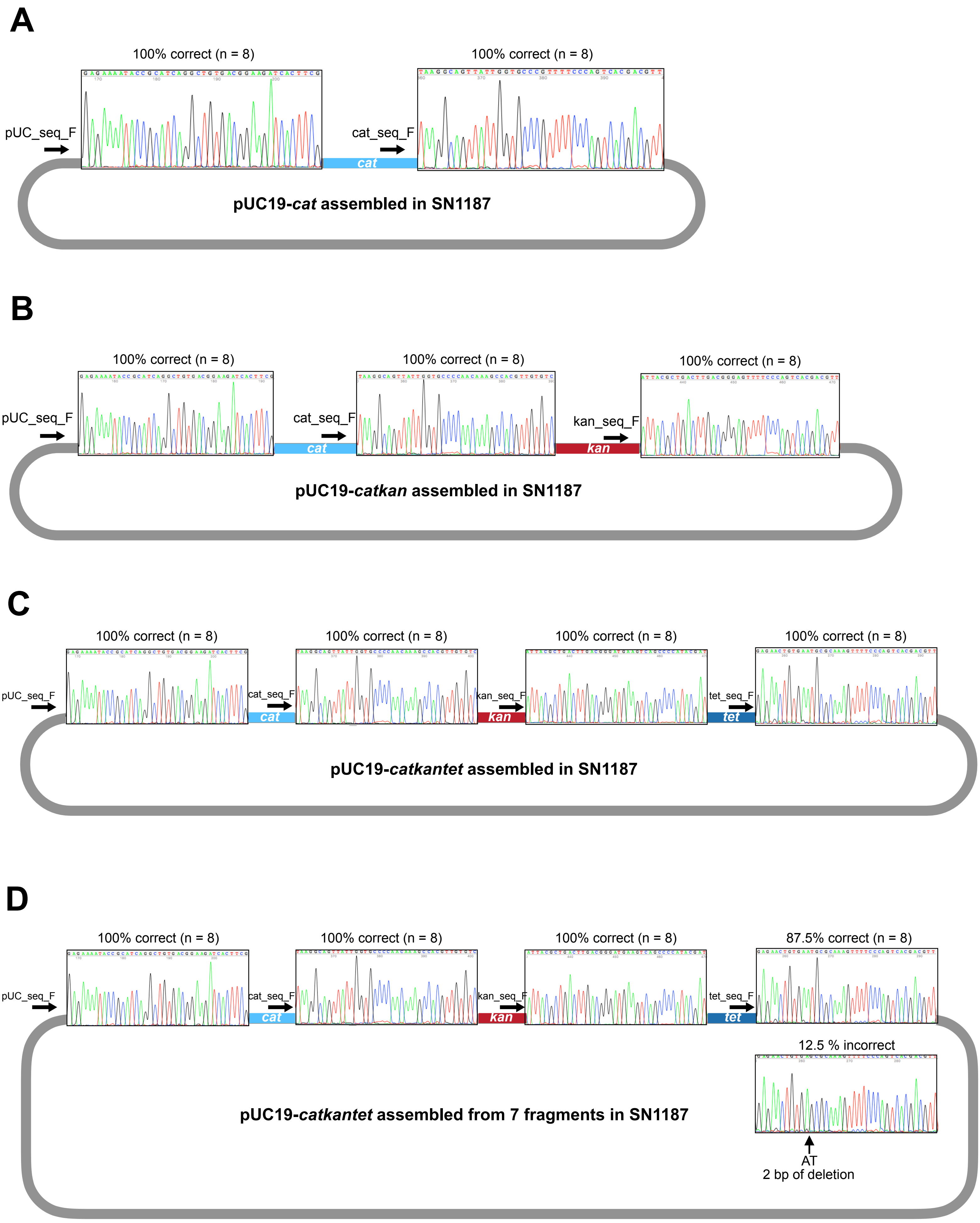
A
100% correct (n = 8)
100% correct (n = 8)
pUC_seq_F
cat_seq_F
cat
pUC19-cat assembled in SN1187
B
100% correct (n = 8)
100% correct (n = 8)
100% correct (n = 8)
pUC_seq_F
cat_seq_F
kan_seq_F
cat
kan
pUC19-catkan assembled in SN1187
C
100% correct (n = 8)
100% correct (n = 8)
100% correct (n = 8)
100% correct (n = 8)
pUC_seq_F
cat_seq_F
kan_seq_F
tet_seq_F
cat
kan
tet
pUC19-catkantet assembled in SN1187
D
100% correct (n = 8)
100% correct (n = 8)
100% correct (n = 8)
87.5% correct (n = 8)
pUC_seq_F
cat_seq_F
kan_seq_F
tet_seq_F
cat
kan
tet
12.5 % incorrect
pUC19-catkantet assembled from 7 fragments in SN1187
AT
2 bp of deletion

Figure S2

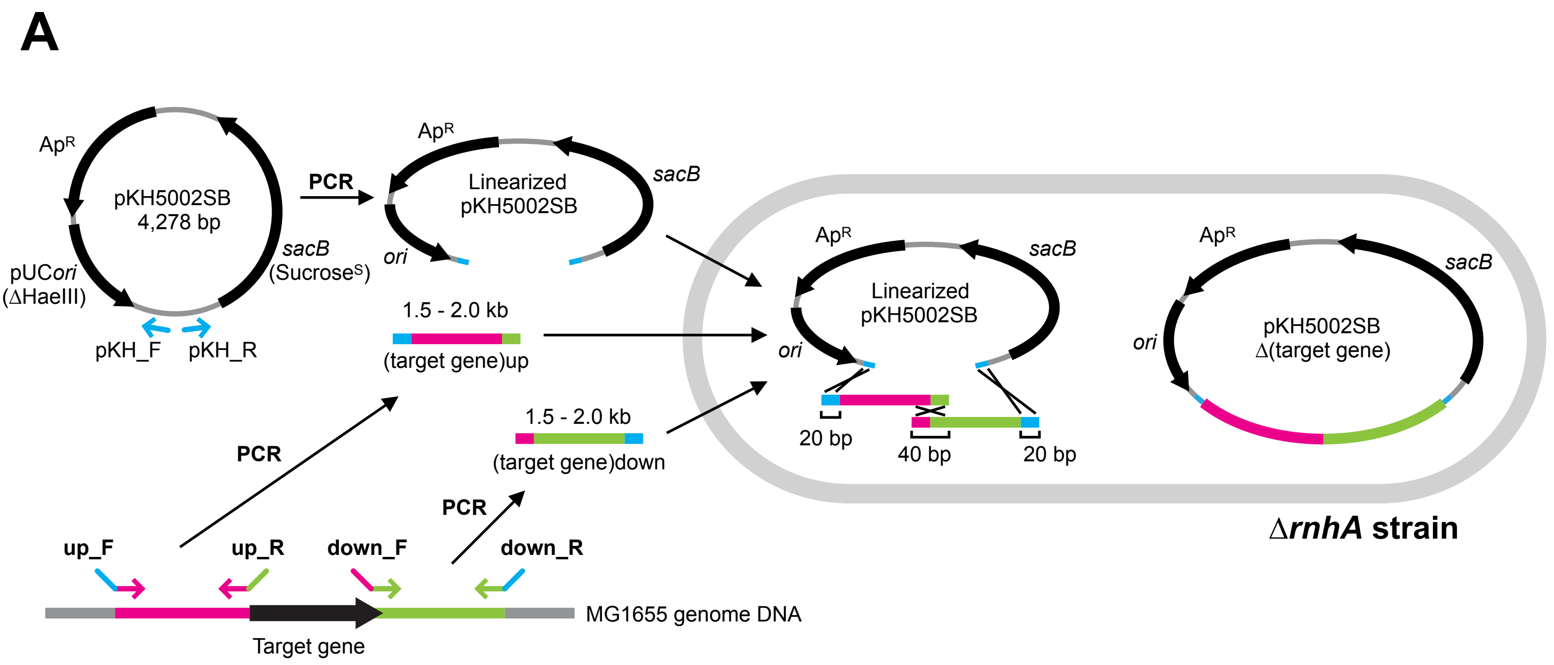
A
ApR
pKH5002SB
4,278 bp
pUCori
(ΔHaeIII)
sacB
(SucroseS)
pKH_F
pKH_R
PCR
ApR
Linearized
pKH5002SB
sacB
ori
1.5 - 2.0 kb
(target gene)up
1.5 - 2.0 kb
(target gene)down
PCR
PCR
up_F
up_R
down_F
down_R
MG1655 genome DNA
Target gene
ApR
sacB
Linearized
pKH5002SB
ori
20 bp
40 bp
20 bp
ApR
sacB
ori
pKH5002SB
Δ(target gene)
ΔrnhA strain

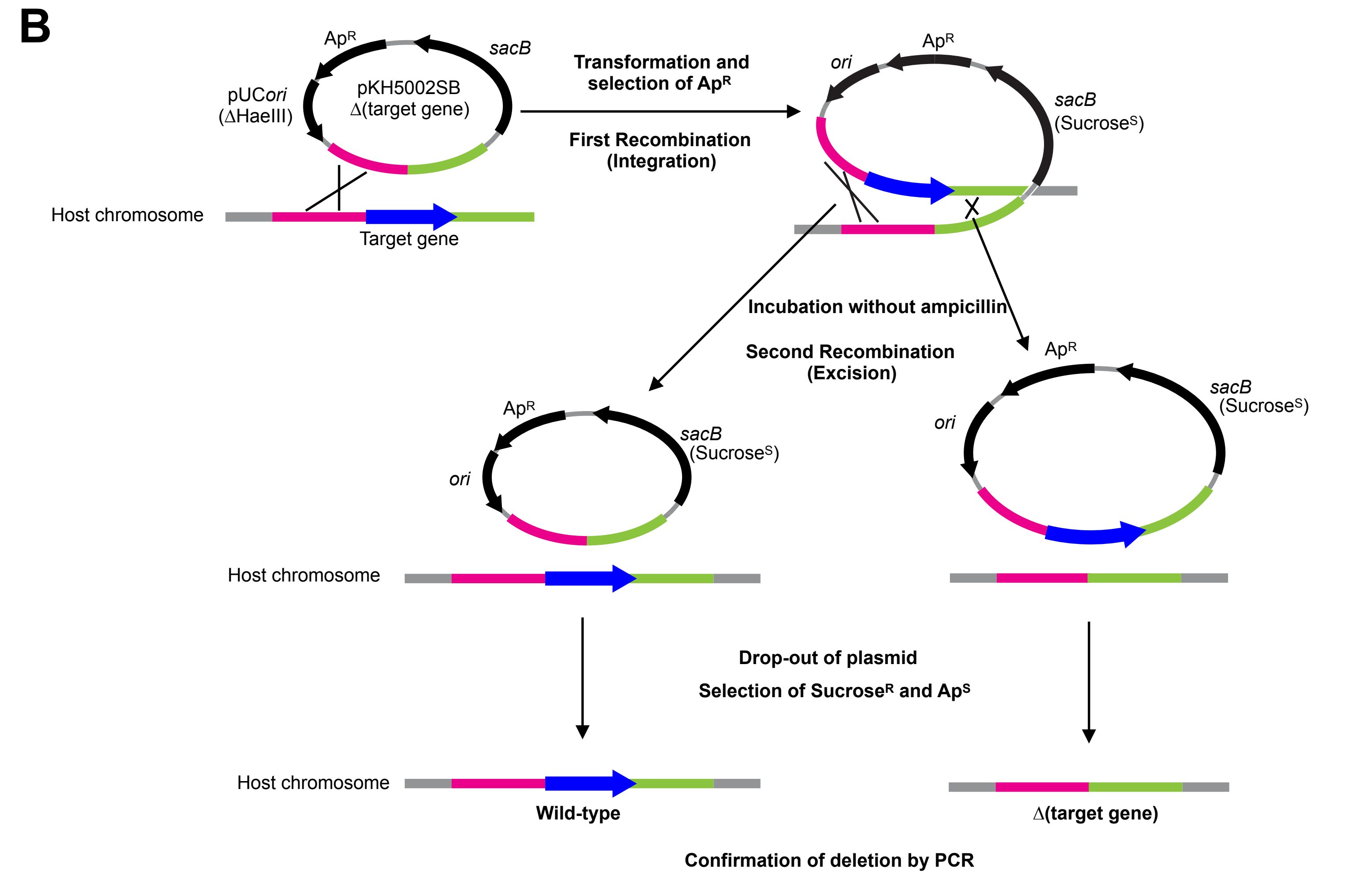
B
ApR
sacB
pUCori
(ΔHaeIII)
pKH5002SB
Δ(target gene)
Host chromosome
Target gene
Transformation and
selection of ApR
First Recombination
(Integration)
ori
ApR
sacB
(SucroseS)
Incubation without ampicillin
Second Recombination
(Excision)
ApR
sacB
(SucroseS)
ori
Host chromosome
ApR
sacB
(SucroseS)
ori
Drop-out of plasmid
Selection of SucroseR and ApS
Host chromosome
Wild-type
Δ(target gene)
Confirmation of deletion by PCR

Fig. S1 Sequencing of the joint region of the assembled plasmids in SN1187.
Eight plasmids of each construct from independent single colonies were analyzed. Primers used for the sequencing reaction and the percentages of correct sequences are shown.

- **A.** Joint sequence of plasmids constructed by the assembly of two fragments with 20 bp homologous overlaps.
- **B.** Joint sequence of plasmids constructed by the assembly of three fragments with 20 bp homologous overlaps.
- **C.** Joint sequence of plasmids constructed by the assembly of four fragments with 20 bp homologous overlaps.
- **D.** Joint sequence of plasmids constructed by the assembly of seven fragments with 40 bp homologous overlaps. A 2 bp region of deletions observed in one of the plasmids is indicated with arrows.

Fig. S2 Construction of deletion mutant by two successive homologous recombinations.

- **A.** Construction of the targeting vector. Linearized pKH5002SB and the upstream and downstream sequences of the target gene were prepared by PCR and assembled in the Δ*rnhA* strain. pKH5002SB could be replicated only in RnaseH-deficient strains, due to deletion of the HaeIII fragment in its replication origin.
- **B.** Deletion of the target gene by two successive homologous recombinations. Since pKH5002SB can be replicated only in RnaseH-deficient strains, the plasmid sequence is not maintained as a plasmid but is maintained in a chromosomally integrated state when the plasmid is introduced into the $rnhA^{+}$ strains. Cells in which the plasmid sequence is integrated into chromosome are selected by ampicillin. *E. coli* cells harboring the *sacB* gene are not viable on an agar plate containing sucrose, and therefore cells in which the plasmid sequence is dropped out are selected on the sucrose plate.
